# Factors influencing establishment of the ovarian reserve and their effects on fertility

**DOI:** 10.21451/1984-3143-AR2018-0011

**Published:** 2018-08-03

**Authors:** Danielle Monniaux

**Affiliations:** UMR Physiologie de la Reproduction et des Comportements, INRA, CNRS, IFCE, Université de Tours, 37380 Nouzilly, France

**Keywords:** cyst breakdown, germ cells, oocyte, ovary, primordial follicle.

## Abstract

A reserve of primordial follicles is set up in the ovaries of fetuses or neonates, depending on the species, and serves as the source of developing follicles throughout the reproductive lifespan. This review focuses on the cellular and molecular mechanisms currently known to control the establishment of this reserve, and their regulation by environmental factors. Most mutations in genes controlling germ cell proliferation and survival, meiosis or follicle assembly lead to the absence of primordial follicles or a sharp reduction in their number, incompatible with fertility in adults. Inadequate maternal nutrition affects the cellular metabolism, increases the oxidative stress and delays follicle formation in fetal ovaries. Despite the existence of compensation mechanisms of some developmental processes, the early-life nutritional environment imprints the long-term ability of follicles to enter growth and develop in adult ovaries. However, maternal undernutrition, overfeeding or high-fat diet during the establishment of the ovarian reserve does not seem to affect the fertility of the female offspring, unless their metabolism or neuroendocrine status is altered. Exposure of fetal or neonatal ovaries to excess steroids inhibits or stimulates follicle formation in a complex manner depending on the nature of the steroid, the dose and the animal species. Estrogens can control follicle formation through intra-ovarian mechanisms involving members of the TGF-beta family such as activin and BMP2. Early-life exposure to synthetic estrogens or environmental pollutants with estrogen-like activity impairs meiotic progression and follicle assembly, and affects long-term primordial follicle activation in adult ovaries. The effects of compounds with estrogen-like activity on the ovarian reserve can be transmitted to several generations through the female germline. Further investigations are needed to establish the early-life effects of the environmental factors on the female reproductive lifespan and decipher the mechanisms of their epigenetic effects on the size and quality of the ovarian reserve.

## Introduction

In the ovary, the growing follicles develop from a reserve of primordial follicles constituted early in life and gradually emptied by both follicle growth activation and follicle degeneration. From this first reserve of primordial follicles, a second ovarian reserve is formed, which consists of gonadotropin-responsive small antral growing follicles and is a dynamic reserve for ovulation. The mechanisms regulating the transitions between reserves have been recently reviewed (Monniaux *et al*., 2014).

When established, the reserve of primordial follicles is oversized in all species. For instance, in the fetal bovine ovary at the end of the first trimester of pregnancy, it comprises millions of follicles (Erickson, 1966), of which only some hundreds of them will grow up to the ovulatory stage during the postnatal life. An intriguing observation is that the size of the ovarian reserve in the early postnatal life varies importantly between individuals of the same species or strain (sheep: McNatty *et al*., 1995; pig: Black and Erickson, 1968; humans: Block, 1952; Baker, 1963; Forabosco and Sforza, 2007). Recently, Ireland *et al* working on cattle reproduction proposed that the maternal environment has a critical role in regulation of the inherent high variation in the ovarian reserve. Moreover, they raised the important question: does size matter in females? and argued that young adults with a low ovarian reserve of primordial follicles have low numbers of growing follicles and phenotypic characteristics usually associated with ovarian aging and infertility (Ireland *et al*., 2011). How the establishment of the first reserve during the fetal or the early postnatal life can affect in a sustainable way the quality and growing features of the follicles of the second reserve, and finally the female fertility in adult life, remains a challenging question, however.

This review will outline the different steps of the establishment of the reserve of primordial follicles and our current knowledge of the molecular and environmental control of this set up. From recent data available in different animal models and in humans, we will try to understand how the environmental factors may regulate the size of the ovarian reserve and the developmental capacity of the primordial follicles, and discuss their possible long-term effects on female fertility and longevity of reproduction.

## Main steps of ovarian reserve establishment

**Figure 1:**
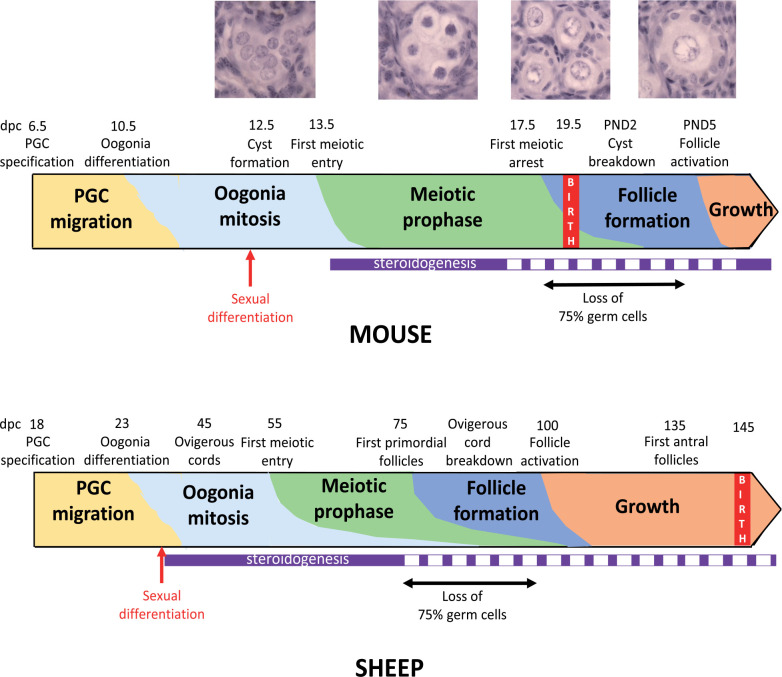
Timeline of the different steps of primordial follicle formation and activation in mouse and sheep ovaries. The periods of ovarian steroidogenesis activity are indicated in purple (full line: high activity, dotted line: low activity). Photographs illustrate the histological appearance of germ and somatic cells from germ cell cyst formation to the primary follicle stage (Monniaux and Brisard, 2018; INRA, Nouzilly, France; unpublished pictures). From Dutta *et al*., 2014; Findlay *et al*., 2015; Grive and Freiman, 2015 for mouse and McNatty *et al*., 1995; Quirke *et al*., 2001; Juengel *et al*., 2002 for sheep. PGC: primordial germ cells; dpc: days post-conception.

The setting up of the reserve of primordial follicles occurs through similar mechanisms in all mammals, but the timing of the processes underlying the formation of the reserve is species-specific (Monniaux *et al*., 2014; Fig. 1). Gonad morphogenesis involves the invasion of the genital ridge by mesonephros-derived cells, which associate with a founder population of primordial germ cells (PGC), as demonstrated in sheep and cattle (Zamboni *et al*., 1979; McNatty *et al*., 2000; Hummitzsch *et al*., 2013). Upon arrival at the gonad, the germs cells, named oogonia at this stage, enter synchronous mitotic divisions with incomplete cytokinesis, forming clonal cell clusters named germ cell cysts or nests, as shown in mouse (Pepling and Spradling, 1998). While the oogonia are dividing to form cysts, they also interact with somatic cells in the ovary. The germ cells and epithelial pre-granulosa cells become organized into ovigerous or ovarian cords, outlined by a basement membrane that separates them from the mesenchymal cells of the developing ovary.

The ovigerous cords are notably well developed in sheep and cattle and they remain until primordial follicles begin to form (Juengel *et al*., 2002; Sawyer *et al*., 2002; Burkhart *et al*., 2010; Garverick *et al*., 2010; Hummitzsch *et al*., 2013). At this stage of intensive mitotic activity of oogonia, the number of germ cells increases exponentially up to about 15 000 per ovary in mouse (Myers *et al*., 2014), 2 700 000 in cow (Erickson, 1966), 500 000 to 1 000 000 in sheep (Smith *et al*., 1993) and more than 5 000 000 in humans (Baker, 1963).

After cessation of mitosis, the oogonia enter meiosis and become oocytes, which progress through the stages of meiotic prophase I arresting in the diplotene stage. Afterwards, the germ cell cysts break apart and individual oocytes become surrounded by granulosa cells forming primordial follicles. It is estimated that during cyst breakdown, about 75% of the oocytes are lost through programmed cell death, including apoptosis and autophagy, as demonstrated in mouse (Pepling and Spradling, 2001; Rodrigues *et al*., 2009).

The functional significance of germ cell development in the mouse cysts was unknown until recently. Using lineage tracing, Lei and Spradling investigated how the mouse germ cells are physically connected to one another and they observed centrosomes, Golgi material, and mitochondria traveling through large gaps in the plasma membrane and accumulating as a Balbiani body in a subset of oocytes during mouse perinatal oocyte development. These Balbiani body-containing oocytes survive and give rise to mature oocytes whereas the oocytes without Balbiani bodies would be only nurse cells as they look smaller, lose most of their cytoplasm, and appear to undergo apoptosis (Lei and Spradling, 2016). Each oocyte is associated with about four nurse cells (Pepling, 2016), that accounts for the dramatic loss of germ cells occurring during cyst breakdown. It appears that this development step determines in a large part the size of the reserve of primordial follicles and their ability to develop.

The formation of follicles proceeds centrifugally from the interface of the cortex and medulla towards the outer region of the cortex, as shown in humans (Konishi *et al*., 1986), rodents (Hirshfield, 1992; Mork *et al*., 2012), sheep (Juengel *et al*., 2002; Sawyer *et al*., 2002) and cattle (Burkhart *et al*., 2010; Hummitzsch *et al*., 2013). Moreover, in the bovine fetal ovary, the ovarian cortex forms lobes appearing to have slightly different developmental ages and giving rise to primordial follicles asynchronously within the same ovary (Burkhart *et al*., 2010; Garverick *et al*., 2010). In mouse, the primordial follicles were shown to consist of two classes that harbor a distinct development path. The medullary primordial follicles are synchronously activated after birth, forming the first wave of activated fast-growing follicles that may aid in the onset of puberty and provide mature oocytes up to 3 months of age. In contrast, the cortical primordial follicles are gradually activated, are slower growing and contribute to ovulation at later stages of the reproductive life (Zheng *et al*., 2014a). The existence of two populations of primordial follicles that exhibit distinct developmental dynamics and contribute differently to ovarian physiology is also suggested in primates (Zheng *et al*., 2014b). Their possible existence in sheep and cattle might explain the bi-phasic pattern of changes in ovarian follicle recruitment and growth observed in these species before puberty (Rawlings *et al*., 2003).

Recent evidence supports the existence of stem cells of a number of the different cell types within the ovary (Hummitzsch *et al*., 2015). Particularly, the isolation of oogonial stem cells from adult mouse and human ovaries has been reported; these cells exhibit both germ and stem cell markers in culture (White *et al*., 2012). When reintroduced into an ovarian somatic environment, the mouse oogonial stem cells have generated follicles capable of producing healthy offspring (Zou *et al*., 2009). However, the oogonial stem cells are unable to sustain by themselves the ovarian function into an advanced age, partly due to age-related changes in the ovarian microenvironment (Truman *et al*., 2017). Moreover, there are no data on their potential physiological role within the ovary, and specifically no evidence that they can contribute to the primordial follicle pool (Grieve *et al*., 2015). The dogma that a fixed pool of primordial follicles formed early in life serves as the only source of developing follicles throughout the reproductive lifespan still holds true (Lei and Spradling, 2013; Zhang *et al*., 2014).

## Molecular control

**Figure 2:**
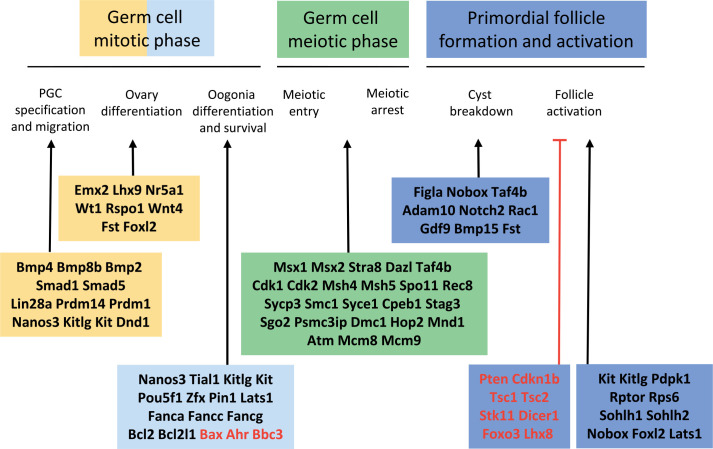
Genes with *in vivo* mutations affecting the different steps of primordial follicle formation and activation in mouse. Genetically modified mouse models exhibit phenotypes similar to those of premature ovarian insufficiency in humans. Black font: positive regulators, red font: negative regulators. From Edson *et al*., 2009; Jagarlamudi *et al*., 2010; Baillet and Mandon-Pépin, 2012; Monget *et al*., 2012; Pelosi *et al*., 2015.

During the last decades, the role of factors controlling the different phases of ovary and follicle formation has been deciphered, thanks to the generation of transgenic mice using gene knock out, knock in, targeted deletion, or over-expression strategies (Edson *et al*., 2009; Baillet and Mandon-Pépin, 2012; Monget *et al*., 2012; Pepling, 2012; Kerr *et al*., 2013; Findlay *et al*., 2015; Grive and Freiman, 2015). Mouse genetic models have identified numerous genes involved in PGC differentiation and migration, oogonia survival and proliferation, as well as in the initiation and execution of meiotic prophase in oocytes (Fig. 2). The mechanisms underlying these processes are generally well conserved from *Drosophila* to mice. The recently established transcriptome and DNA methylome landscapes of human migrating and gonadal PGCs were found in general similar to those of mouse PGCs at comparable stages (Guo *et al*., 2015). Owing to this high degree of conservation, factors shown to affect ovarian reserve establishment in mouse models are all potential candidates for identifying mutations associated with premature ovarian insufficiency in humans (Jagarlamudi *et al*., 2010; Pelosi *et al*., 2015), and with infertility in domestic animal species. However, despite conserved principles between species, some mechanistic differences exist between mice and a range of species including pigs, monkeys and humans, as exemplified for PGC specification at early developmental stages (Tang *et al*., 2016; Kobayashi *et al*., 2017). In this review, in addition to the knowledge acquired from mouse models, data from various species are presented and compared where possible.

As said above, primordial follicles are formed by germ cell cyst breakdown and assembly of individual oocytes with pre-granulosa cells. Intricate regulation of gene expression, including the oocyte-specific Figla (Soyal *et al*., 2000), Nobox (Lechowska *et al*., 2011) and Taf4b (Grive *et al*., 2014) transcription factors, is critical for these processes in mouse. The oocyte-specific secreted factors BMP15 and GDF9, known to drive the growth of small follicles and the maturation of the cumulus-oocyte complex during folliculogenesis (Monniaux, 2016), might participate also in controlling follicle formation. Indeed, the formation of multi-oocyte follicles, indicative of the presence of defects in cyst breakdown and oocyte assembly with pre-granulosa cells, has been observed in Bmp15-/- Gdf9+/- mice (Yan *et al*., 2001). Moreover, it was recently reported that GDF9 and BMP15 can induce the formation of follicles from human embryonic stem cells expressing the germ cell-specific proteins DAZL and BOULE (Jung *et al*., 2017). However, the role of GDF9 and BMP15 in follicle formation *in vivo* remains speculative in humans and other species, particularly because BMP15 is not expressed until the follicle is actually formed and begins to grow. In the fetal sheep ovary, *GDF9* expression starts at 56 days post-conception (dpc) and is located in oocytes (Mandon-Pépin *et al*., 2003; Juengel *et al*., 2004). However, *BMP15* expression is hardly detectable before 94 dpc, and in sheep homozygous for the *FecX^I^* inactivating mutation in *BMP15*, follicle formation seems normal (McNatty *et al*., 1995). Some observations suggest that BMPR1B, a BMP receptor known to bind BMP15/GDF9 heterodimers and *BMP15* homodimers, may still participate in follicle formation in sheep. In the fetal sheep ovary, *BMPR1B* expression starts as soon as 25 dpc, is high at the time of germline cyst breakdown (Mandon-Pépin *et al*., 2003) and is located in the mesonephric-derived cell streams and the ovigerous cords (Reader *et al*., 2012). In the ovaries of fetuses and newborn lambs homozygous for the *FecB^B^* mutation in *BMPR1B*, the oocytes of the primordial follicles are larger and contain a greater volume of mitochondria, smooth endoplasmic reticulum and ribosomes, suggesting that the mutant form of BMPR1B has influenced the process of germline cyst breakdown and led to follicles better equipped for the initiation of follicular growth (Reader *et al*., 2012). The role of BMPR1B in the mechanisms of follicle formation and the BMP ligands able to activate BMPR1B signaling at this stage in the sheep ovary remain to be defined. Nevertheless, it can be speculated that BMP15 secreted by the first growing follicles may activate BMPR1B signaling in the ovigerous cords and influence the subsequent formation of follicles.

In addition, some somatic cell-derived factors play important roles in cyst breakdown. Indeed, estradiol signaling is critical for inhibiting this process until birth in rodents (Chen *et al*., 2007) and may regulate it in ruminants as well (Fortune *et al*., 2010, 2013). In mouse, growth factors such as activin/follistatin and neurotrophins also influence cyst breakdown and the size of the primordial follicle pool (Bristol-Gould *et al*., 2006; Kerr *et al*., 2009; Kimura *et al*., 2011), and recently the disintegrin Adam10 has been shown to govern the recruitment of the pre-granulosa cells in cysts (Feng *et al*., 2016). From data in mice, the actions of these various factors converge to modulate the Jagged/Notch signaling pathway in germ cell cysts. Particularly, the Notch2 gene encoding a Notch receptor in pre-granulosa cells orchestrates cyst breakdown, postnatal apoptosis of oocytes and primordial follicles assembly (Xu and Gridley, 2013) and the oocyte-specific secretory proteins Jagged1 (a Notch ligand), Gdf9 and Bmp15 activate the Notch signaling pathway in pre-granulosa cells (Zhao *et al*., 2016). All these observations highlight the importance of crosstalk between germ cells and pre-granulosa cells for the formation of primordial follicles.

The inactivation of genes controlling the processes of PGC specification and migration, ovary differentiation, meiosis initiation and execution of meiotic prophase leads generally to the absence of germ cells in the ovaries of mutant mice. Most inactivation of genes involved in the regulation of oogonia proliferation and survival, or germ cell cyst breakdown lead also to female infertility due to the absence of primordial follicles or a sharp reduction in their numbers at birth. However, in some cases, as in the inactivation of the pro-apoptotic factors *Bax* and *Bbc3*, or the over-expression of the pro-survival factor *Bcl2*, the number of primordial follicles of the initial reserve is increased (Perez *et al*., 1999; Flaws *et al*., 2001; Myers *et al*., 2014). In mice over-expressing *Bcl2* in ovaries, the surfeit of primordial follicles is not maintained in the long term, suggesting that the ovary may contain a sensing mechanism by which excess numbers of primordial follicles at birth are detected and removed from the ovary by adulthood (Flaws *et al*., 2001). The importance of postnatal regulations of the number of primordial follicles has also been shown in ewes carrying the *FecB^B^* mutation in *BMPR1B*. The establishment of the ovarian reserve is delayed in mutant ewes (McNatty *et al*., 1995) and at birth their ovaries contain lower numbers of primordial follicles than wild-type ewes, but at 5 years of age mutant ewes are still fertile and their ovarian reserve is even higher, due to a lower rate of primordial follicle activation (Ruoss *et al*., 2009). These examples in mouse and sheep genetic models demonstrate that, in the presence of a mutation affecting moderately the establishment of the initial reserve of primordial follicles, the fertility and reproductive longevity of the female are not determined at birth. Similar compensatory postnatal mechanisms have been observed following the administration of activin to neonatal mice, since despite an increased number of primordial follicles in the mice ovaries after treatment, the excess follicles containing oocytes of poor quality is eliminated prior to puberty (Bristol-Gould *et al*., 2006).

## Environmental and hormonal control

In sheep, as in cattle and humans, primordial follicles are formed before birth (Fig. 1) so that the establishment of the ovarian reserve is under the control of the maternal environment. All metabolic, hormonal or health changes in the maternal compartment and pollutants able to cross the placenta may affect the development of the fetal gonads. These maternal or external environmental factors can act directly on the developing ovary, but they impact also on various organs, particularly the hypothalamo-pituitary complex, the liver and the pancreas (Rhind *et al*., 2001; Padmanabhan and Veiga-Lopez, 2013), as well as the adipose tissue, all able to affect indirectly ovarian function and fertility. Whether the environmental factors acting directly on follicle formation can also affect long-term fertility, and through which mechanisms, is a difficult question. This review will focus on the known effects of nutrition, steroids and some endocrine-disrupting factors on ovarian reserve establishment, and discuss their mechanisms of action and potential consequences for fertility.

## Nutritional factors

Maternal nutritional status participates in programming growth, development, and function of the major fetal organ systems. Placental insufficiency impairs fetal development and reduces the number of primordial follicles in the ovaries of fetuses and neonates in humans (de Bruin *et al*., 1998) and sheep (Da Silva *et al*., 2003). Maternal overnutrition reduces also the number of primordial follicles in fetal bovine ovaries at the end of pregnancy, but concomitantly the number of growing follicles is increased, suggesting that the initiation of follicular growth has been activated (Weller *et al*., 2016). There is little information on the effects of maternal nutrition on the initial size of the pool of primordial follicles, but increasing evidence demonstrates that nutrition modulates the dynamics of ovarian reserve establishment in ruminant fetuses and rodent neonates, with functional consequences on follicular growth.

Undernutrition of ewes from the time of mating significantly retards ovarian development in fetal ovaries. Particularly, the ovaries of fetuses from feed-restricted ewes contain more germ cells entering the initial stages of meiosis at a time when a large proportion of them has completed this process (Borwick *et al*., 1997). This delay in meiosis onset is induced by the combination of maternal undernutrition before ovarian differentiation (days 0-30 of pregnancy) and during the phases of germ cell mitosis and meiosis entry (days 31-65 of pregnancy; Rae *et al*., 2001). Interestingly, undernutrition imposed during each of these gestational periods reduces also the incidence of follicle development beyond the primordial stages (Rae *et al*., 2001). These observations indicate the existence of a precocious window of ovarian sensitivity, during which nutritional disorders can affect the dynamics of follicle formation and compromise subsequent follicular development.

The mechanism of action of nutrients on follicle formation remains poorly understood. Female rats born to mothers fed a high-fat diet throughout pregnancy have fewer oocytes in fetal ovaries at embryonic day 20 and it was speculated that increased maternal-fetal inflammation associated with maternal obesity (Aye *et al*., 2014) may have contributed to accelerated fetal oocyte loss (Tsoulis *et al*., 2016). In a murine pharmacological model of maternal diabetes induced by streptozotocin administration, *in utero* exposure of mice to hyperglycemia decreases the expression of genes involved in meiosis initiation (*Stra8, Dmc1* and *Sycp3*) and germ cell cyst breakdown (*Nobox, Figla* and *Bmp15*) and impairs the initiation of meiosis and follicle assembly in the fetal offspring ovaries (Qiu *et al*., 2017). The starvation of mouse pups between 1.5 and 3 days of postnatal life, when most primordial follicles are assembling, leads also to a decrease in the expression of *Nobox* and the impairment of germ cell cyst breakdown; in the ovaries of the starved pups, the alteration of metabolic parameters, exemplified by the lower expression of genes encoding proteins of fatty acid synthesis such as *Fabp5, Cpt2* and *Acsl3*, could have triggered an oxidative stress responsible for the increased autophagy and apoptosis observed in the oocytes within cysts and in the primordial follicles (Wang *et al*., 2017).

**Figure 3:**
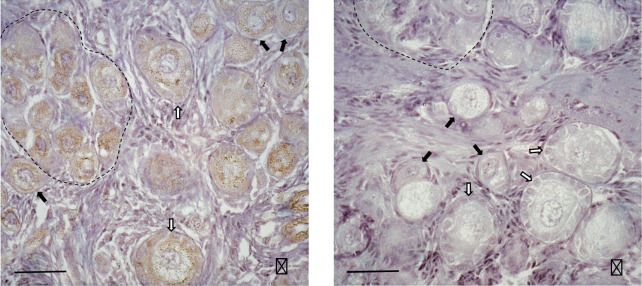
Expression of leptin receptors in the ovaries of 21 days-old piglets. For immunostaining, ovarian sections were incubated with leptin receptor antibody (Ob-R (M-18)-R, sc-1834-R; Santa Cruz Biotechnology Inc, Heidelberg, Germany; dilution 1/250), without (A) and with (B) displacement by the peptide used as immunogen for antibody preparation. Leptin receptors are detectable in all germ cells and some granulosa cells of primary follicles. Dotted lines delineate germ cell cysts. Primordial and primary follicles are indicated by black and open arrows, respectively. Bar = 50µm. (Monniaux and Brisard, 2018; INRA, Nouzilly, France; unpublished pictures).

Besides a possible direct effect of glucose and other nutrients on ovarian development, metabolic hormones can regulate follicle formation. Insulin accelerates primordial follicle assembly in rodent ovaries and increases apoptosis in germ cell cysts *in vitro*, as shown in hamster (Yu and Roy, 1999) and mouse (Feng *et al*., 2015). Leptin, a major adipokine able to modulate lipid and glucose metabolism and insulin sensitivity, could also regulate follicle formation. Indeed, in the ovaries of piglets containing a substantial proportion of germ cells not yet enclosed in follicles but grouped into germ cell cysts, leptin receptors are present on oogonia and oocytes (Fig. 3). These germ cells were found to express six different isoforms of leptin receptors, potentially able to activate the MAP kinases and PI3/AKT pathways (Attig *et al*., 2013). Moreover, postnatal leptin treatment of piglets with intra-uterine growth retardation accelerates follicle formation and activation, since the ovaries of leptin-treated piglets contain lower percentages of oogonia and oocytes in meiotic prophase, but higher percentages of oocytes in the dictyate stage within germ cell cysts, primordial and primary follicles (Attig *et al*., 2013).

Whether the early-life nutritional environment affects fertility and reproductive longevity of mammals is a controversial issue (Gardner *et al*., 2009; Sloboda *et al*., 2011). After induction of maternal diabetes by streptozotocin in mouse, the reproductive performance of the offspring is severely decreased in the long term, but their postnatal growth and physical development are also impaired or delayed, indicating the establishment of a sustainable metabolic syndrome (Spadotto *et al*., 2012). In sheep, inadequate maternal nutrition has little effect, if any, on puberty and ovulation rate of the offspring when postnatal growth is normal (Da Silva *et al*., 2001; Rae *et al*., 2002). None of the studies of maternal undernutrition, overfeeding or high-fat diet during the period of establishment of the ovarian reserve of their offspring have demonstrated that the fertility or reproductive longevity of the offspring is affected in the absence of metabolic syndrome.

Some changes in germ cell numbers induced by undernutrition or overfeeding in the early phases of ovarian development are compensated in offspring at birth or after a period of normal feeding. For instance, in rats born to mothers fed a high-fat diet throughout pregnancy and lactation, despite a significant decrease in oocyte number during late fetal life, neonates at day 4 of postnatal life demonstrate higher numbers of primordial follicles than control offspring, suggesting that a recovery mechanism has occurred during follicle formation (Tsoulis *et al*., 2016). Also, following a reduced rate of germ cell cyst breakdown induced by the starvation of mouse pups in early postnatal life, the animals recover a normal number of primordial follicles in their ovaries after 3 weeks from re-feeding (Wang *et al*., 2017). Despite the existence of compensation mechanisms in developmental processes, the early-life nutritional environment before or/and during primordial follicle formation leaves an imprint since it modulates the long-term ability of follicles to enter growth and develop. Indeed, neonatal overfeeding of rats induces the accelerated activation of primordial follicles before puberty and at adulthood (Sominsky *et al*., 2016). Moreover, maternal high-fat diet induces follicular atresia in the ovaries of rabbit and rat offspring in adulthood (Leveillé *et al*., 2014; Tsoulis *et al*., 2016). Interestingly, in the rat model, antral follicles of adult offspring exhibit reduced FSH responsiveness and express low levels of the estrogen receptor *Esr1* and the oocyte secreted factor *Gdf9* (Tsoulis *et al*., 2016), both factors well known to regulate follicular development and FSH sensitivity. Furthermore, undernutrition of rats during pregnancy decreases the number of antral growing follicles in the ovaries of adult offspring (Bernal *et al*., 2010). In a similar way, offspring from cows nutritionally restricted during their first trimester of gestation (before the full establishment of the ovarian reserve in offspring) exhibit, from birth to the adult age, lower numbers of antral follicles than control offspring from non-restricted mothers (Sullivan *et al*., 2009; Mossa *et al*., 2013). This difference in folliculogenesis activity between offspring from restricted and non-restricted cows cannot be attributed to metabolic differences since offspring have similar birth weights and postnatal growth rates (Mossa *et al*., 2013). Cows with low numbers of antral follicles on their ovaries are known to have numerous phenotypic characteristics usually associated with subfertility (Ireland *et al*., 2011), but whether alterations in folliculogenesis of the offspring after precocious maternal nutritional restriction may, in turn, impact on their fertility and reproductive longevity has not been assessed in this bovine model.

## Steroids and endocrine-disrupting factors

In cattle and sheep, fetal ovarian capacity to produce steroids is high before follicle formation begins and decreases around the time follicles first appear (Quirke *et al*., 2001; Yang and Fortune, 2008; Fig. 1). In mice, fetal ovaries have also a significant steroidogenic activity, which drops before cyst breakdown; in addition, they are exposed to high levels of maternal steroids, which fall dramatically after birth (Dutta *et al*., 2014). From these observations, the hypothesis that steroids may inhibit follicle formation was tested *in vitro* on postnatal rodent ovaries and fetal bovine ovaries. In rat (Kezele and Skinner, 2003), mouse (Chen *et al*., 2007) and cattle ovaries (Nilsson and Skinner, 2009), progesterone inhibits follicle formation, whereas the non-aromatizable androgen 5alpha-dihydrotestosterone does not in cattle ovaries (Fortune *et al*., 2010). Estradiol at high concentrations impairs primordial follicle formation in mice and cattle (Chen *et al*., 2007; Fortune *et al*., 2010) but not in rats, whereas conversely low concentrations of estradiol stimulate primordial follicle formation *in vivo* and *in vitro* in hamster ovaries (Wang and Roy, 2007). *In vivo*, the number of primordial follicles is reduced by 50% in the ovaries of near-term fetal baboons deprived of estrogen by administration of an aromatase inhibitor *in utero*, and restored to normal in animals supplemented with estrogen (Zachos *et al*., 2002). Moreover, fetuses of sheep androgenized *in utero* from day 30 of pregnancy have, at day 90, nearly double the proportion of germ cells enclosed in follicles compared with control animals (Comim *et al*., 2015), indicating that follicle formation is accelerated by testosterone or by estrogenic action stemming from aromatization of testosterone to estradiol. Altogether, these observations indicate that each steroid acts upon follicle formation in a complex species-specific and dose-dependent fashion.

In sheep, the receptors of estrogens (ESR1 and ESR2), androgens (AR), as well as progesterone (PGR), are all expressed in the surface epithelium and ovarian stroma of the fetal ovaries (Juengel *et al*., 2006). In cattle, ESR1 is mostly found in the surface epithelium whereas ESR2 is expressed in the medulla, germ cells and pre-granulosa cells during early fetal life, then both receptors seem to be expressed in all cell types after 110 dpc (Burkhart *et al*., 2010; Garverick *et al*., 2010). In humans, ESR2 is localized primarily to germ cells, but AR expression is confined to somatic cells between clusters of germ cells (Fowler *et al*., 2011). The potential exists therefore that all steroids could interfere with somatic-to-germ cell signaling during follicle formation, but it needs exploring. Interestingly, estrogen receptors, particularly ESR2, are expressed in all types of germ cells and in pre-granulosa cells in sheep, cattle and humans, suggesting that both direct and indirect actions of estrogens are possible on germ cells during follicle formation. From the data available, estrogens could control follicle formation through intra-ovarian mechanisms involving members of the TGF-beta family. Indeed, estrogens regulate the formation of primordial follicles in baboons by controlling the intra-ovarian inhibin/activin ratio (Billiar *et al*., 2003) and recently, BMP2 has been shown to mediate the effect of estrogens on follicle formation in the hamster ovary, since the interference of BMP2 production or its receptor function disrupts estradiol-stimulated primordial follicle formation (Chakraborty and Roy, 2017). However, the mechanism by which low and high concentrations of estrogens exert opposite effects on follicle formation are not yet understood.

Increasing evidence indicate that environmental factors with estrogen-like activity modulate importantly follicle formation. Mice treated neonatally with genistein, the primary soy phytoestrogen, have multi-oocyte follicles in ovaries; genistein was found to inhibit germ cell cyst breakdown and attenuate oocyte cell death, and these effects are mediated by the estrogen receptor Esr2 (Jefferson *et al*., 2006). Similar effects were observed after exposure of neonatal mice to synthetic estrogens, such as diethylstilbestrol, ethinylestradiol and bisphenol A (Karavan and Pepling, 2012). Oral administration of zearalenone (a mycoestrogen produced by *Fusarium graminearum*) to pregnant mice impairs germ cell meiotic progression, decreases the expression of the meiosis-specific genes *Dazl, Stra8, Scp1* and *Scp3*, increases DNA double-strand breaks at the diplotene stage and affects primordial follicle assembly in the fetal ovaries (Liu *et al*., 2017). In a similar way, following exposure of pregnant mice to diethylhexylphthalate (DEHP), a widespread plasticizer with estrogen-like activity, the first meiotic progression of female fetal germ cells is delayed, associated with an increase in DNA methylation level of *Stra8* and a decrease in its expression levels (Zhang *et al*., 2015). In neonatal mice, DEHP impairs also primordial follicle assembly, while decreasing the gene and protein expression of Esr2 and components of Notch signaling in mouse ovaries (Mu *et al*., 2015). In sheep, exposure (by grazing pastures fertilized with sewage sludge) of pregnant ewes to a real-life mixture of environmental chemicals with pro-estrogenic actions disrupts fetal ovarian development and alters the fetal ovarian transcriptome and proteome at day 110 of pregnancy (Fowler *et al*., 2008).

Estradiol is not required for the initiation of follicle growth in mice (Britt *et al*., 2000) but aromatase knockout mice have reduced numbers of primordial and primary follicles compared with wild-type mice at 10 weeks of age (Britt *et al*., 2004). The primordial follicles that form in ovaries of estrogen-deprived baboon fetuses contain oocytes with a marked reduction in microvilli, structures essential for uptake of substrates from surrounding granulosa cells and presumably long-term follicle survival (Zachos *et al*., 2004). However, lowering estrogen levels during the period of follicle formation does not impair folliculogenesis and ovulation at adulthood in baboons (Pepe *et al*., 2013).

Steroid excess during follicle formation can be deleterious for subsequent follicular development. The exposure of ewes between 60 and 80 days of pregnancy to a mixture of environmental chemicals with estrogen-like activity decreases primordial follicle activation in fetal ovaries near term and increases atresia rate in activated follicles (Bellingham *et al*., 2013; Lea *et al*., 2016). In contrast, prenatal testosterone excess between days 30 and 90 of pregnancy enhances the activation of primordial follicles and early follicular development in fetal and postnatal sheep ovaries (Steckler *et al*., 2005; Smith *et al*., 2009). In sheep, fetal exposure to excess testosterone was shown to disrupt the ovarian proliferation/apoptosis balance, with a decrease in BAX expression in the primordial and primary follicles of fetuses appearing to be programmed by androgenic actions, and changes in PCNA, BCL2, and CASP3 expression in the growing follicles of adults programmed by estrogenic actions of testosterone (Salvetti *et al*., 2012). This prenatal treatment by testosterone leads to early reproductive failure in adult ewes (Clarke *et al*., 1977; Birch *et al*., 2003), resulting from the combination of neuroendocrine, metabolic and ovarian defects (Padmanabhan and Veiga-Lopez, 2013) and similar conclusions were drawn from rodent models of early exposure to steroids (Zambrano *et al*., 2014).

Early exposure to environmental factors with estrogen-like activity affects long-term primordial follicle activation and the observed effects are transgenerational. For instance, maternal DEHP exposure significantly accelerates the recruitment of primordial follicles in the F1 and F2 generations of mice (Zhang *et al*., 2015). The modification of the DNA methylation of imprinted genes in F1 mouse oocytes induced by maternal DEHP exposure is heritable to F2 offspring (Li *et al*., 2014). Recently, it was shown that the reduction of the ovarian follicular reserve and of the oocyte developmental capacity induced by maternal DEHP exposure is transmitted through the female germline up to the third generation (Pocar *et al*., 2017).

## Conclusions

**Figure 4:**
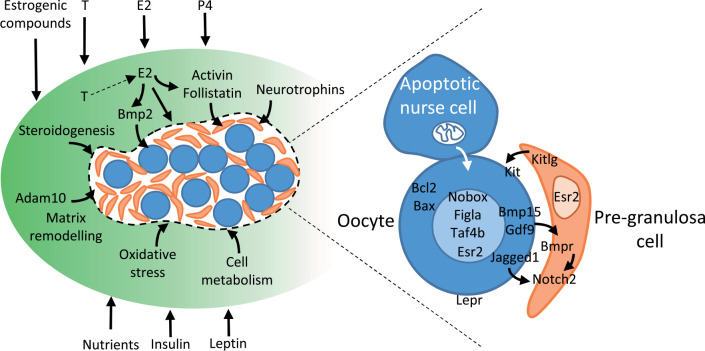
Schematic representation of some mechanisms currently known to regulate germ cell cyst breakdown and follicle assembly. A germ cell cyst is represented (delineated by a dotted line), containing oocytes (blue round cells) and pre-granulosa cells (small pink cells). The intra-ovarian mechanisms regulating germ cell cyst breakdown and the environmental factors currently known to modulate them are indicated in the left part of the figure. Some known interactions between germ and somatic cells, and between germ cells themselves, are zoomed in the right part of the figure.

During the last decades, our knowledge of the cellular mechanisms and the molecular and environmental control of the establishment of the ovarian reserve of the primordial follicles has made significant progress. Some of the intra-ovarian mechanisms currently known to control germ cell cyst breakdown and primordial follicle assembly are illustrated in Fig. 4. Inadequate maternal nutrition, exposure to steroid excess or to environmental pollutants with estrogen-like activity at the time of follicle formation can delay or modulate the mechanisms controlling the establishment of the ovarian reserve, leading to the formation of a decreased number of primordial follicles in the fetal or neonatal ovaries.

The size of the initial follicular reserve is primarily dependent on genetic determinants, and mutations affecting factors controlling the different steps of ovarian reserve establishment can lead to the absence of primordial follicles, or a sharp reduction in their numbers, incompatible with fertility in the adult. However, having two- or three-fold more or less follicles than the mean number characteristic of the species at birth is not a determinant for fertility and reproductive longevity in adulthood, as demonstrated in mice (Flaws *et al*., 2001) and sheep (Ruoss *et al*., 2009). Rather, the dynamics of follicle consumption from the initial primordial follicle pool determine the female reproductive lifespan (Monniaux *et al*., 2014).

From the data available, the ovarian changes induced by environmental factors present during the fetal or neonatal life do not seem to alter the fertility of young females, unless their metabolism or neuroendocrine status is affected, but their consequences on the reproductive lifespan have not yet been established. To understand the impact of the early-life environment on fertility of both young and older adults, there is now a significant need to consider the different types of populations of primordial follicles, which have distinct developmental dynamics and contribute differently to ovarian functionality (Zheng *et al*., 2014b).

The early-life ovarian environment modulates in the long term the rate of primordial activation and the dynamics of the small growing follicles in adult ovaries. As these processes are little or not influenced by the gonadotropins, the observed changes result likely from direct imprinting effects of environmental factors on the germ and/or somatic ovarian cells, occurring before and during follicle formation. Increasing evidence indicate that environmental chemicals with estrogen-like activity leave epigenetic marks on the germ cells, and their effects are transmitted through the female germline up to the third generation in mice (Pocar *et al*., 2017). Further investigations are needed now to decipher the underlying mechanisms of these epigenetic effects and understand the long-term consequences of gene imprinting on the size and quality of the ovarian reserve.
